# A Review on the Role of the Neuroscience of Flow States in the Modern World

**DOI:** 10.3390/bs10090137

**Published:** 2020-09-09

**Authors:** Joshua Gold, Joseph Ciorciari

**Affiliations:** 1 Centre for Mental Health, Swinburne Neuroimaging (SNI), Swinburne University of Technology, P.O. Box 218, Hawthorn, Melbourne, VIC 3122, Australia; jciorciari@swin.edu.au; 2 Department of Psychological Sciences, Swinburne University of Technology, P.O. Box 218, Hawthorn, Melbourne, VIC 3122, Australia

**Keywords:** flow state, productivity, expertise, neurocognition, transcranial direct current stimulation, decision-making

## Abstract

Flow states have been shown to help people reach peak performance, yet this elusive state is not easily attained. The review describes the current state of literature on flow by addressing the environmental influences as well as the cognitive and neurocognitive elements that underlie the experience. In particular, the research focusses on the transition of cognitive control from an explicit to an implicit process. This is further expanded upon to look at the current, yet related neurocognitive research of high performance associated with the implicit process of automaticity. Finally, the review focusses on transcranial direct current stimulation (tDCS) as a novel method to facilitates an induction of flow states. Implications are aimed at a general technique to improve on skill acquisition and overall performance.

## 1. Introduction to Flow

The scientific community has as of late begun to explore the field of expertise and its components. One element however that has begun to gain a growing amount of attention is the peak performance found in flow states, whether it be in sport, business or other professional endeavors. Flow is described as a state of optimal performance denoted by smooth and accurate performance with an acute absorption in the task to the point of time dissociation and dissociative tendencies [1,2,3]. In the modern workplace there are so many distractions, from messages to meetings, that result in a reduction of productivity. Yet a 10-year longitudinal study Cranston and Keller [4] showed people in flow states were 500% more productive. Whilst much research has been performed on the personality components of flow there is still much to explore when it comes to the neurocognitive underpinnings of flow to better understand the workings and catalysts for this elusive state. This review focusses on describing the current state of flow research on neurocognitive understandings and provides an insight into the key theories and experimental implications being presented in the research surrounding flow states.

Transcendent, spiritual experiences similar to flow states have long shared reports with countless of religious references dating back centuries by spiritual authors. Flow then found its entrance into the mainstream with Maslow [5] ‘peak experiences’ and has since been appropriated into popular culture with many names including “in the zone” and “in the moment”. Although a long history exists of this high functioning state, much of its inner workings and route of initiation is shrouded in mystery. Csikszentmihalyi [6] first described the flow state and noticed the conditions for entering this experiential state include a balance of challenges or action opportunities with an individual’s skill as well as clear and well-defined goals with immediate feedback.

**Figure 1:**
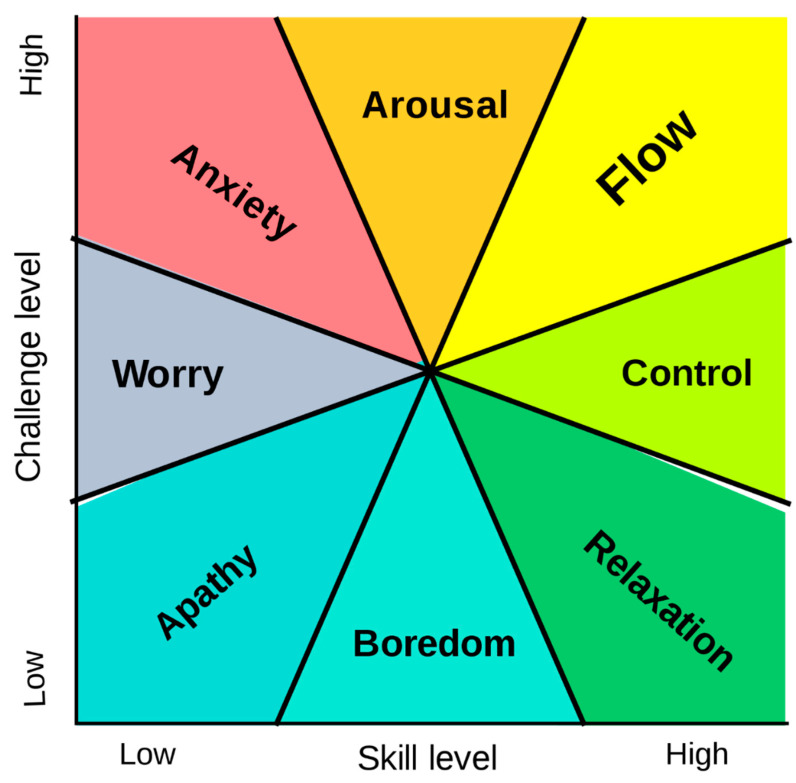
Csikszentmihalyi’s flow model [7] describes psychological states in terms of challenge level and skill level.

According to Csikszentmihalyi [7] flow theory, the flow experience relates to the skill set perceived to be possessed by the individual relative to the perceived challenges of the activity. Challenges can be considered as “opportunities for action” thus flow is produced by any situation that requires skill [3]. The phenomenology of flow further suggests that the enjoyment of a task is due to a discovery found within the interaction of the task. For instance, at first the task might appear boring or anxiety provoking but if the action opportunities become clearer or the skill level improves the task becomes more engaging and finally enjoyable. The discovery of more complex behaviors results in an emergent motivation that transforms a previously unengaging task into that which is intrinsically motivating [8]. Therefore, complexity of the skill must increase to meet the increasing complexity of the task’s challenge in order for the person to remain in flow. Csikszentmihalyi [7] developed the flow state model to help illustrate this state change as seen in Figure 1. For instance, when the challenges and skills are low, a person will likely experience apathy, considered an experience of the lowest quality and the lowest intensity on the flow state model. Whereas, when the skills are greater than those needed for the challenges, the person is more likely to experience boredom/ relaxation, considered an experience of higher quality than apathy. As the level of challenge increases, the experience moves toward control. In contrast to this, when challenges are greater than the skills required by the person, the experience of worry/ anxiety is more likely. Then as the skill level increases, the experience moves toward arousal. Therefore, based on this model, flow states are believed to be accessed when skills and challenges are both high and in equilibrium, resulting in an experience of the highest quality [9].

Nonetheless, flow rarely occurs in everyday life because challenges and skills are rarely balanced, but even these two parameters do not guarantee flow. Therefore, flow requires activities to have a further set of particular criteria [9]. Firstly, the activity typically requires learning of skills, and have clear goals with quick and unambiguous feedback. This affords a sense of control over reality by understanding what needs to be done and how they are performing. This activity design also works best when concentration and involvement is facilitated by separating a person from their everyday existence by focusing on the particular reality of the activity, such as particular uniforms and special rules of the activity that are not necessarily relevant to everyday living [9].

People in flow mention that they become so absorbed in the activity that they do not have any attention to spare to become distracted by anything else. People have also mentioned a collection of other psychological phenomena associated with states. These include: (a) a feeling of control over the activity; (b) an experience of time distortion, in which a person loses awareness of how time is passing (c) the removal of self-consciousness in which a person loses the awareness of themselves as well as thoughts of everyday problems; (d) a feeling of transcendence where the person feels a sense of unity with the activity. See Table 1 for a list of full 9 components.

Therefore, when a person has a perceived adequacy of skills matched with above average challenges, as part of a goal-directed, rule bound system that provides clear feedback, the person can find complete absorption that removes the possibility of any distractions from thoughts irrelevant to the task at hand. In this focused space a person has an opportunity to find such a level of immersion in the activity that they will feel an inspired sense of control, a complete removal of self-consciousness, a distortion of time and a feeling of transcendence.

Furthermore, it has also been found that flow states can be reached by any person performing any sort of task as long as they can ascertain an adequate level of skill. These levels of skill require an expertise that can afford the smooth performance state associated with flow and consequently with higher expertise is believed higher flow values [10]. Many people were studied in many different situations and all were able to achieve the optimal experience from the activity. Flow states have become such common place in all areas of society that people use many ways of describing the state such as “wired in”, “in the groove”, “in the moment” and “the zone” to name a few. This experience has typically been described throughout the ages as forms of religious fervor but now has moved into the current day through many other forms of engaging activities. Flow has been recorded in everything from business transactions, sports, video gaming, music, art and yoga. These flow states all share in a series of similar characteristics that were attributed to flow by Csikszentmihalyi. It is the subjective challenges and skills, not the objective ones, that impact on the quality of a someone’s experience [8]. Numerous studies have further highlighted the similar subjective experience of flow states in various activities, such as sport [11], gambling [12], skateboarding [13], education [14] to name a few. No matter what the activity, the elicitation of this flow state is considered by many to be the “Holy Grail” of performance [15].

### 1.1. Environmental Influences on Flow

Even when one has satisfied the conditions stipulated as necessary to reach the flow state, this however still does not conclusively answer how certain people are able to reach this state nor why and whether all people are able to attain such a state [16]. One element noted by Csikszentmihalyi that influences entrance into flow states is the level demanded by the critical implications of the activity [9]. This has been shown to ascribe to a normative continuum to the flow experience based on the task’s personal importance. For example, surgery and mountain climbing are highly critical tasks, which are more often reported to result in intense, ecstatic flow experiences whereas absorbing yet less critical tasks such as reading, and video games have less intense flow experiences.

Additionally, flow states have been shown to be moderated by the level of perceived importance a person places on a task. A study by Engeser and Rheinberg [17] showed that importance impacts the skill/ challenge requirements. During activities considered important such as exams, flow was high when the challenge was low while activities considered less important such as playing Pac-Man, flow was highest when there was a skills/ challenge balance but low when the challenge was too low or high. Additionally, this study showed the importance of achievement motives, based on the risk taking models of Atkinson [18], who showed how the explicit motive of fear of failure and the implicit motive of hope for success influenced the preference towards a balance of challenge and skill. In particular, people with the hope for success are more likely to experience flow during balanced skills/ challenge task compared to individuals high in fear of failure who experience less flow when balanced.

In considering these additional implications of criticality, importance and achievement motives, these lead to the introduction of environmental aspects such as the role of the task. For instance, how do these elements apply to work compared to recreational task? A study by Csikszentmihalyi and LeFevre [19] showed surprisingly that flow was three times more likely to occur during work than recreation. However, even within work it depends on the role. For instance, managers reported the highest levels of flow in work while general workers reported the highest level in recreational flow. Furthermore, a recent study by Viljoen [20] of part time and professional musicians on the experience of flow looked at these elements to show the differences in their approach to the task. Occupational musicians showed a significant connection between mindfulness and frequency of playing which is associated to accessing flow. Yet, part time musicians found that a professional musician’s occupation became routine and likely inhibited flow. To clarify, mindfulness has described as a connecting bridge between our mind and the present moment, allowing the person to stay aware of what is happening in that very moment [21]. Therefore, mindfulness appears to share similar attributes that may support flow state facilitation. Additionally, the struggle of financial security for occupational musicians placed stress on many musicians which also was distracting from achieving flow states. Therefore, it is worth considering the difference of recreational and occupational roles and the related levels of task frequency regarding perceived expertise when measuring flow states.

When delving further into what flow is and how far reaching and common flow states are in modern society, it is also important to understand why flow is so relevant to modern day society. In the modern workplace, there are so many opportunities to be distracted from work with messages, meetings and social media, it is difficult to not become distracted or overwhelmed. When in a flow state, the individual is considered to perform at their full capacity [22,23]. Flow has commonly been associated with intense concentration [9], a higher behavioral efficiency and creativity [24], and heightened sense of playfulness [25]. Furthermore, the intrinsic rewards associated with autotelic experience is likely to increase learning efficiencies [24], as well as better remembering of the experience and also more likely to seek such experiences more often [9]. This helps drives the person to ever-higher levels of complexity in the challenge of the activity, ultimately improving their skill level. Such increases have also been shown to impact positively on the associated group with many successful scientists, sports stars and artists mentioning flow as relevant to their work and improving their performance whether it be in sports, arts or workplace productivity [9]. Flow is also characterized by an elevated sense of self-control [26] and higher positive subjective experiences [7].

### 1.2. Flow Measurement

The primary method of studying flow has been through questionnaires as well as interviews for more qualitative explorations. For example, Larson and Csikszentmihalyi [27] developed the experience sample method (ESM) which would ask participants to mark in real time at certain times throughout their day of their flow experience. The problem with this and other methods is, as was already stated, flow states require acute concentration to the point where little to no attentional resources is misallocated. Also, the individual experiences a loss of self-consciousness where self-reflective thoughts and fear of social evaluation are not present. Therefore, the introspection necessary for these measuring techniques has the danger of inhibiting the flow experience as it requires resources to be allocated to a different cognitive set as these are retrospective by nature [28].

Since ESM, the Flow State Scale (FSS) was introduced, which operationalizes flow by transforming it’s nine elements into dimensions that load equally on a composite flow score [29]. The FSS considers flow as a ‘degree’ of flow on a continuum instead of a discrete ‘peak’ experience, which can be used to portray the experiential quality as a level of intensity of flow within the activity [3]. The intensity of the flow experience is considered to elevate as more of the nine elements increase in score. The FSS is typically given at the end of a task in order not to force the participant out of the state during the task, however, people will experience a range of affective states across trial periods [30]. Self-reported flow experience scales at the end of a task measure the experience across the whole task rather than for a particular time period. This may further be influenced by the recency effect in memory which may color the memory of the entire trial by the most recent experience toward the end of the trial [31]. Such pitfalls of studying the dynamics underpinning flow states limit how far researchers explore this elusive state to optimal performance and our understanding of consciousness. Researchers have since begun to address this limitation through the use of psychophysiological methodologies, which focus on the expression of psychological phenomena in bodily processes, to explore the dynamic nature of flow experience throughout the entire task.

Psychophysiological measures—such as hypnosis, meditation and sleep—have been employed to explore the more complex physiological aspects of human consciousness. These measures include electrocardiography (ECG), electromyography (EMG) and skin conductance and have begun to be utilized in the study of flow states. More recently electroencephalography (EEG) and functional magnetic resonance imaging (fMRI) are shedding new light on the neurocognitive elements of flow states. However, these studies have not accumulated enough evidence to define a common acceptance of the neurocognitive functioning and thus researchers continue to address flow states from different methodological backgrounds and these different motivations result in differing perspectives. Select studies have now begun to measure flow states and demonstrate the psychological effects of flow with physiological results utilizing various physiological variations to justify experimental design decisions, yet this results in conflicting results from multiple research queries. Therefore, during this exploratory phase, precise research questions are still ill-defined due to the disparate results.

### 1.3. Flow Neurocognitive Mechanisms

As flow research has continued to delve deeper into its neural functioning, theorists have naturally moved to explain the neurocognitive mechanisms underpinning the state. Dietrich [32] proposed a flexibility/ efficiency trade off, which addresses the balance between implicit and explicit processing systems used to acquire, memorize and represent knowledge [33]. Theories of implicit/ explicit processing has been guided by the modern understanding on neuroscience which assumes a more hierarchical development of cognitive functions where an increase of integrated neural structures continues to increase the level of complex processing. Therefore, Dietrich [32] introduced the first neurocognitive model for flow states as the transient hypofrontality hypothesis (THH) which considered flow a state of transient downregulation of the highest cognitive hierarchical component, the prefrontal cortices, defining flow processes in the form of transition from explicit to implicit information-processing systems.

The majority of research [34] have found a consensus about the nature of the explicit system, different to the implicit system, as a rule based system linked to language function and conscious awareness across many tasks, e.g., the serial reaction time task [35], the dynamic control task [36] and many others. However, there is still contradictory evidence found in the explicit reasoning ability of Köhler’s apes [37] despite their presence of language. Nonetheless, whilst verbalizability appears to be the general standard, a better theoretical principle is still needed for conscious awareness. Conscious awareness has been described along similar lines as the implicit and explicit system with both on and offline systems that work to establish consciousness. Systems offline to consciousness are reflexive, rigid and fast responding, such as a frog snapping at a fly. However, as it is organizationally inefficient to house the ever-increasing number of complex reflexes, a more effective system proposed would be to include a temporary buffer that enables the organism to examine multiple representation of the plan of action before making a decision [38].

Conscious online elements appear to share a close relationship with working memory and executive control. Executive control directs our attention and the working memory. It also links the past, present and future by providing a moment-to-moment permanence. Findings on the association between the prefrontal cortex with this prevailing model was developed by Crick and Koch [38] which states that conscious awareness can only exist if the brain activity projects to the prefrontal cortex. Crick and Koch’s theory however is not a complete theory of conscious awareness and therefore we are relegated to using the operational definition that explicit processes are able to be explained verbally [38].

Studies have started identifying the prefrontal regions involvement in the explicit system due to evidence of the dorsolateral prefrontal cortex (DLPFC) acting both as a working memory buffer for the content of consciousness, as well as selecting content through the executive attentional network [39,40]. The medial temporal lobe structures have also been identified as relevant underlying circuitry [41]. An argument has been presented that the explicit system is a more recent evolutionary occurrence and present in animals with more highly developed prefrontal areas [42]. Support is found for this in the late phylogeny and ontogeny development of the prefrontal cortex [43]. Furthermore, the structure of information processing is known to be hierarchical and due to the sophistication of the explicit knowledge representation, such higher order structures are believed to be localized in the prefrontal areas [42].

Two distinct parallel processing tracts have been identified that traverse the brain and process the incoming information differently. The emotional tract processes more typically in a non-algorithmic skill-based manner that attaches values to help evaluate the biological significance of the information. The second tract performs detailed featured analysis in a computational mode free from any interpretations of salient information. Whilst both pathways begin to converge at the thalamus, the cognitive pathway feeds through the hippocampal formation and temporal, occipital and parietal cortices (TOP), helping provide a degree of selective attention required to process incoming information [44].

As connections continue to take place along the hierarchical pathways, full convergence appears to occur at the Dorsolateral Prefrontal Cortex (DLPFC) [45]. The DLPFC is primarily involved in executive functioning by enabling higher functionality such as self-reflective consciousness, abstract thinking, and theory of mind [46]. Furthermore, it plans, formulating appropriate strategies and subsequently directs the motor cortices to initiate the process. It is at these prefrontal functioning in which the control over the cortices are the most sophisticated. The DLPFC is also responsible for temporal integration [47], directed and sustained attention [48], and working memory [45], which facilitate an intricate cognitive framework that actively attends to information, thus affording a buffer to hold such information in mind, whilst organizing it in space-time [40].

This cognitive tract is broken up into two attentional tracts of its own: Top down and bottom-up processing. As explained by Corbetta and Shulman [49], voluntary shifts of attention are thought to be mediated by the dorsal frontoparietal system resulting in goal-directed, “top-down” signals arising from knowledge about the current activity such as finding your way home. On the other hand, the ventral frontoparietal system mediates the automatic “bottom-up” capture of attention guided by salient properties inherent in the stimuli, such as your unique and alarming ringtone. The DLPFC has been shown to exhibit a top-down functionality which inhibits maladaptive and inappropriate cognitive and emotional behavior [50]. The frontal lobes appear to be based on more universal principles which inhibit people from compulsively acting on immediate cues [51]. Therefore, the frontal lobes help free us from slavery to direct environmental triggers. It is the inhibitory abilities of the top-down processes that allow a person to remain task focused and not be guided by more salient bottom up processes [52].

### 1.4. Neurocognitive Models of Flow

The two predominant neurocognitive theories of flow states have helped guide flow research to better understand its function in order to be able to further support access and entry into flow. The first, transient hypofrontality hypothesis (THH) by Dietrich [32] proposes that during flow states, these explicit executive functions of the frontal cortices are inhibited. This reduction of frontal activity is expected to reduce interference from explicit processing such as self-referential thought and thereby freeing up more resources to be dedicated to the faster implicit processing system such as actioning of automatized processes. Recently studies have begun using psychophysiological measures to test the THH of flow experiences with a variety of testing for Electroencephalography (EEG) with shooting [53], arithmetic [54], video games [55] and memory tasks [56], as well as Functional Magnetic Resonance Imaging (fMRI) for arithmetic tasks [57,58]. For instance, Hirao [59] conducted a near-infrared spectroscopy (fNIRS) on occupational therapy students who completed a verbal fluency test. Whilst there were only 2 channels in the study (FP1/2), the results supported the THH in which a negative correlation was associated between higher flow states resulting in a suppression of prefrontal activity.

However, the synchronization theory of flow (STF) proposed by Weber and Tamborini [60] disputes the THH due to many flow-like activities such as hypnosis and meditation showing strong frontal activity in neuroimaging studies occurring when in these altered states of consciousness as well as in flow studies [61]. Therefore, STF instead focusses on the neuronal efficient, feature binding processes of synchronizing neurons and networks to more effectively communicate and create “holistic, higher-order experiences” that resemble flow states.

STF’s foundation is based on Posner et al., [62] tripartite theory of attention that focuses on the neurocognitive structures of attention including the frontal and parietal cortices relating to “alerting” (the process of becoming aware of a stimulus), the top-down componentry of the dorsal attention network including the superior and inferior parietal lobes, the frontal eye fields, and the superior colliculus for “orienting” (allocating attentional resources to a stimulus), and the prefrontal “executive” regions for goal-directed processing. A few studies to date have provided support for STF [63,64,65], with one of the first fMRI studies by Klasen et al. [66], who broke down a video game into five operationalized elements of flow that can be observed as characteristics of the activity to find activation in relevant attention and reward structures that support the STF.

Whilst there are fundamental differences in both THH and STF, they do share a similar belief in the role of the emotional tract in managing automatization of implicit processes as well as intrinsic reward. Implicit categorization has found less agreement with these theories than what has been found for the explicit system. While the role of explicit knowledge in consciousness is thought to create a more behaviorally flexible global workspace to test hypotheses [67], the role of implicit knowledge is believed to be more task-specific and thus less flexible due to the difficulty to access from other parts of the system [68].

One thing agreed on is that the implicit system is not accessible to the conscious awareness [54,69]. However, unlike memory theorists [70] (e.g., Schacter) who hold that ‘implicit’ implies no conscious awareness of the details or even that a memory was stored, a weaker criterion is used for category learning, which only requires that the nature of learning has no conscious access [34]. However, there may be an awareness of some learning occurring because trial-by-trial feedback is typically present. For instance, when a participant receives feedback that their action was correct, they will understand and be conscious of a learning having taken place. Implicit categorization theory is relevant to flow experiences as the literature has stated such states require clear and timely feedback. When feedback has been removed people are then restricted to verbalized rules [34].

There has been a lot of research also supporting the implicit system as experience or skill based and conveyed through performance rather than verbally [39]. Implicit categorization learning was shown in a study by Spiering and Ashby [71] to provide optimal training results when the challenge level of the task begins with difficult examples and then move to easier examples after it is understood that no simple verbal rule is sufficient. Rather than getting locked into a verbalized single rule, implicit learning allows decision making to take a more integrative approach from different perceptual dimensions. This information integration approach is maximized only at the pre-decisional stage as two or more stimulus components are integrated [72].

While it has typically been assumed that an exemplar-similarity-based system should dominate information-integration tasks [73,74], COVIS instead assumes a procedural-learning system. COVIS, an acronym for “competition between verbal and implicit systems”, which describes the process of the verbal system dominating initially due to the strength of its connections but with task repetition the implicit system supersedes the explicit verbal system bias. Yet both systems remain active retaining a significant proportion of categorization judgments after learning is complete [72].

Although the neural substrates are less clear for the implicit system, the basal ganglia (BG) have most often been critically associated with implicit system [41]. The BG are interconnected masses of gray matter positioned in the interior regions of the limbic cortices and in the upper part of the brainstem. This key region of the BG receives all extrastriate visual cortex projections, with about 10,000 visual glutaminergic connections to each caudate cell in the striatum [75]. Projections are then sent to various cortical premotor and prefrontal regions via two synaptic pathway convergences. The first synaptic connection is via the globus palladus and substantia nigra pars reticula which has dopaminergic connections while the second synapse of the ventral anterior nucleus of the thalamus projects off to premotor areas, specifically Brodmann’s Area 8 [76].

COVIS places an emphasis on this synaptic convergence as a critical site of procedural learning [77]. In particular, it appears there are three factors which contribute to cortical-striatal strengthening via long term potentiation (LTP): strong presynaptic; and postsynaptic activation; as well as dopamine release [78,79]. While presynaptic and postsynaptic activations are considered to play an important role for LTP via stimulus driven high threshold sensory cortical cells [80], dopamine is considered more as a reward-mediated training signal [79]. These synapses however weaken through long term depression if either postsynaptic activation or dopamine release is not present [78]. This could occur for example if an incorrect response is given resulting in an absence of dopamine release or if only a weak response is recorder by the visual cortical cell. Maddox et al., [81] showed support for an interesting prediction by this three-factor model that if feedback was delayed by more than 2.5 s then information integration learning would be severely inhibited. This appears to lend support to the notion that flow states may require timely and accurate feedback whereas explicit learning in rule-based tasks of equal difficulty could sustain delays.

Implicit systems are believed to process parallel tasks due to the limitations of bandwidth that exist in the working memory of the explicit systems [82]. Cowan presented evidence of the working memory capacity with a 4 ± 1 limit, after rehearsal and chunking were catered for [83]. Therefore, the explicit system appears to be capacity limited, where information demands are too great parallel tasks are collapsed into fewer chunks deeming some information inaccessible [82]. Implicit systems on the other hand seem to not share the same limitations. When learning a new task such as driving a car, this is a multidimensional task with many elements working in parallel. While the cortex is considered to be utilized for managing the input of novel information due to the requirement of goal directed attention and flexibility, working memory typically would be overwhelmed as instructions typically involve more than four independent bits of information. Therefore, instructions could be broken down into smaller components that could then be combined into larger chunks once the skill is sufficiently acquired. The explicit instructions would form a mental representation of the task that requires the premotor, primary motor and parietal cortex, as well as the cerebellum, to execute it [84]. Because of the limited ability to combine items into chunks, learning slows down due to capacity restrictions. The BG are believed to be a passive observer during this time building its own representation of the action [85]. After sufficient practice, neural control is gradually shifted to the BG [86] and also the supplementary motor cortex, motor cortex, thalamus, and hippocampus [84]. Ultimately, this internalizes the pattern of this activity into “muscle memory” and thereby affords the BG primary control without much reliance placed on the prefrontal explicit regions [87]. This internalization frees up computational space of the executive function for other activities such as observing the surrounding environment, due to a lessening of demand from working memory. This may be useful for flow as it frees the person from needing to focus on the skill of the task and gives more buffering room for anticipating the potential challenges of the task.

Furthermore, a basic level of skill acquisition is needed to have a flow experience, as the implicit system requires a series of learnt specialized and independent response patterns to output [3]. These automated stimulus response procedures are believed to require many hours of highly dedicated practice. Learning of automated responses takes time because of the limited ability of the explicit working memory to transfer specialized and reflexive response patterns to the implicit system due to capacity restrictions [32,86]. Automaticity, in which thoughts and behaviours occur without the need for conscious guidance, can be both conscious and unconscious [88]. Unconscious automaticity are defined as automatic processes that do not require any willful initiation and operate independent of conscious control [89]. This is exemplified with a priming that biases further processing of an event without the person necessarily even consciously aware of the connection. Such as seeing a beer advertisement along with a hot day and suddenly realizing you are thirsty and want a beer.

Conscious automaticity are automatic thoughts and behaviours that provide efficient implementation of an action by providing faster processing through the removal of conscious monitoring as well as the use of minimal attention capacity [89]. The modern standard for determining automaticity is if the behaviour can be produced in parallel and without attention [88]. Skill acquisition is generally conscious labored and slow but becomes automatic with consistent and frequent practice. These mentally disparate processes are then repackaged into a fluid arrangement of actions that can be set off by a single thought [90]. Furthermore, automaticity enables assumptions to be made based on experience which creates greater outcome predictability. The more a person monitors their intentions throughout their actions, the more their experience will be consciously willed and nonautomatic.

A key component of the BG, the dorsal striatum, has been associated with the role of automatized implicit learning, in particular, both STF and THH models have shown strong support not only for automatization of implicit functions but also dopaminergic influence in which fMRI flow studies have exhibited BG and striatal activation [58,59,60,61,62,63,64,65,66], along with increased striatal dopamine during flow states in support of the role of implicit control of BG [91,92]. The striatum’s volume increased after a skill acquisition period of a video game task [93]. It is also assumed that the striatum is an early development in human evolution because of its central location and action as an input nucleus for the BG. The caudate nucleus of the striatum has also been shown as the primary input structure of the procedural learning system using the COVIS model [77]. It is long known that to effectively multitask two things simultaneously requires one task to be implicit [94]. Thereby, implicit systems are ultimately more efficient than their explicit counterpart. People that have entered flow states often refer to an automatic processing in which they report task focused behaviour without conscious thinking which suggests a form of frontal inhibition required for successful entry into the state.

Furthermore, a key site of pleasurable experience of rewards is associated with the dopamine rich striatum, due to dopamine’s role in rewarding behavior by predicting rewarding outcomes that would result in reward-seeking experiences [95], which lends support to the autotelic nature of flow states. Because of the autotelic nature and high criticality of flow, we are moved to consider the role of novelty as relevant to the induction of flow states. In the novelty hypothesis, during a period of high criticality, when a person is exposed to a new situation that results in a challenge that is equal to the skill level, the person may be pushed up into a level in which their skill is just below the level of challenge being presented. This additional stimulation may be enough to absorb the final amount of explicit buffering systems in order to fully immerse the performer in the task.

## 2. Exploration of Flow Functions

As flow states are considered a complex combination of multiple cognitive features it has been difficult to delineate specific neurocognitive markers. Studies for the most part still rely on a mix between psychophysiological measures and probing post-task self-report questionnaires. The conflict still remains that as soon as the participant is asked about their experience, they are forced to self-reflect which will move them out of the flow state. We can therefore begin to break down some of the key neurological elements to test whether they can be defined as key elements of flow states in order to further identify key elements that may be relevant to the neurocognitive functionality of flow states. Particular elements of flow to be defined are that it occurs within an activity which is balanced with an individual’s abilities, whilst fully immersed in the task and self-referential thoughts are completely inhibited. However, we can look at previous studies looking at similar cognitive functions such as expert performance, creativity, focused attention and mental workload to help delineate neurocognitive landmarks that will help us identify the elements of flow activity.

The EEG is a well validated measure for examining psychological states during skilled motor performance [96,97]. In particular, results have highlighted the left frontal and temporal regions as playing key roles in expert performance with increased alpha power in EEG occurring in expert marksmen compared to novice shooters [96,97,98]. EEG has also been used across a range of activities including weightlifting [99], golf [100] and archery [101] all revealing a reduction in left hemispheric activity. In a recent study, a comparison of neuro-anatomical characteristics also showed that expert divers have significantly increased cortical thickness in the left superior temporal sulcus compared to the non-athlete group [102]. The superior temporal gyrus houses several important cortical structures, including Wernicke’s area known to be involved in the comprehension of language. To follow on, this pattern of increased alpha activity in the left temporal region has been most commonly interpreted as representing a reduction in cortical activations, reducing verbalizations associated with the left brain and enabling more resources to be allocated to the visual-spatial processes of the right brain [103]. This has been further supported by lower coherence estimates of left temporal regions with motor regions by expert marksmen [104]. This pattern suggests less cortico-cortical communication and a suppression of analytic processing influence thus simplifying a complex process and alleviating the need for a division of cognitive resources.

Additionally, a key antecedent of flow utilizes the challenge/skills-balance which indicates a state of high mental workload from deep involvement in the task [9]. This has been shown in psychophysiological studies on flow, in which decreased heart rate variability was shown during challenge/ skills-balance in a knowledge task [105]. EEG has also been used to evaluate mental workload in which a reduction of alpha activity and an increase of theta is present due to the tasks increased difficulty levels [106,107]. Alpha frequencies are categorized into three frequency bands (8–13 Hz, 8–10 Hz, and 11–13 Hz). Alpha activity in general (8–13 Hz) represents lower levels of consciousness and awareness, while an alpha reduction results in increased mental activity [108]. The low alpha band (8–10 Hz) is associated with the mechanisms of arousal, attention and effort as well as general cognitive processing while high alpha (11–13 Hz) selectively acts according to the encoding of the stimulus [109].

Sports performance has also been shown to improve when implementing hypnotic techniques using flow state suggestions [110,111,112]. It is not yet understood how hypnosis increases performance or the experience of flow. One suggestion by Crawford and Gruzelier [113] is a shift is made from an analytical think style to become more holistic after hypnosis, allowing access to processes that are important for athletic performance. Shifts from the left (analytical verbal and conscious side of the brain) to the right hemisphere (holistic, nonverbal, imaginative side of the brain) have been shown during hypnosis [114]. It has been further shown that there are strong correlations between hypnosis with absorption [115]. A correlation has also been shown between absorption and dissociation, in which the ability to become absorbed in a task is another way to induce dissociative control [116]. Task absorption and dissociation are considered key component to the higher levels of the flow phenomenology.

Additionally, theta activity has been shown as relevant for evaluating cognitive processing during flow like tasks such as meditation. Lutz et al. [117] experienced meditators and novices were tested at the beginning and end of a three-month meditation retreat, using an attentional blink test. In experienced meditators, results significantly improved whilst presenting increased theta phase-locking, i.e., a reduced variability of theta phases across trials. These results are considered to show a more stable execution of neural processing [118]. Furthermore, multiple fMRI studies have highlighted attentional networks providing support for increased activity in prefrontal networks during focused [119], meditation-like attention [120].

Positive affect and motivational orientation, two elements associated with flow phenomenology, have also found links to changes in frontal EEG asymmetry [121]. In particular, increased left alpha frontal activation was correlated with approach-related motivation [122]. This is also shown by higher activity of the frontal left associated with trait measures of behavioral activation [123]. Specifically, positive emotions were correlated with high left frontal activity, while negative emotions were correlated with increased relative right frontal activity [124]. Ultimately a pattern of the relationship between frontal EEG asymmetry, motivational direction, and affective valence has been shown for performance settings.

## 3. Flow Facilitation

To further test the neurocognitive mechanisms of different states and in particular flow states, technologies such as transcranial direct current stimulation (tDCS) have been utilized to provide a clearer understanding on the underlying processes. TDCS is a non-invasive form of brain stimulation that alters cortical excitability based on the direction of current flow at subthreshold levels of the neuronal membrane potential. Anodal stimulation has been shown to increase cortical excitability over the region of electrode placement, while cathodal stimulation inhibits the region’s neuronal excitability. The level of neuronal activity modulation depends on the current density, which is governed by elements such as current strength and electrode variability. Furthermore, the length of after-effects is dependent on stimulation duration. i.e., excitability effects have been shown to last up to 60 min [125], yet results have also shown effects fading after 30 min of stimulation [126].

While tDCS has been used for clinical settings such as depression, Parkinson’s disease and pain management, it has also shown to improve performance in normal participants including working memory [127], visuo-motor learning [128], and categorical learning [129]. Many experimental paradigms have been implemented on motor learning including the more frequently used: skill acquisition and adaptation [130]. New motor skill acquisition involves the ability to execute new motor abilities that improve performance beyond previous levels. Skill acquisition can take weeks or months while skill can decrease due to a lack of ongoing practice. Strategies that improve skill acquisition and retention can be of great scientific and practical interest. For instance, Clark & Coffman [131] showed a unique use of anodal tDCS over parietal and frontal regions for improving skill acquisition speeds by enhancing performance in threat detection within natural scenes that are typically relevant for the effective management of many skills throughout our everyday and specialized work tasks.

Adaptation for sensorimotor tasks, unlike skill acquisition, addresses a new framework of well learned movements and spatial goals instead of requiring new capabilities of muscle activations to be updated. While adaptation can be assisted with explicit control processes, it can also update entirely implicitly [132]. Functionally, adaptation focusses on an error decrease by changing challenge levels to facilitate a return to the previous level of performance, while participants movements are updated due to changes in motor outputs or sensory inputs. TDCS has been shown specifically to enhance adaptation of real world cognitive multi-tasks by specifically targeting the goal-directed dorsal attention network by right parietal anodal stimulation and thereby resulting in improved task performance [133]. It is important to acknowledge that tDCS has been shown to result in ceiling effects for experts compared to novice performers in which at a certain level of expertise, tDCS has been shown to not have a significant impact on performance [134,135,136,137].

More recently tDCS has begun to be implemented to explore its potential role in facilitating flow. In a recent study, Ulrich et al., [138] facilitated higher flow scores for people experiencing low flow after stimulating them during an arithmetic task with a prefrontal (fpZ) anodal tDCS to target the medial prefrontal cortex (MPFC). This is an interesting result as Ulrich et al., [57] showed support for the THH with a deactivation of MPFC during an fMRI study on flow, interpreted as a reduction of explicit functionality of self-referential activity, yet only the excitatory anodal tDCS over the prefrontal regions resulted in an enhancement toward flow states. This was uniquely for people specifically experiencing low flow in which more research is needed for the general population. A tDCS study by Gold & Ciorciari [139] explored flow for novice gamers who typically didn’t find during videogames and expert video gamers who did. The tDCS set up focused on an anodal right parietal and cathodal left frontal stimulation that also showed support for flow state induction, this time in alignment with a deactivation of MPFC associated with high flow states. Therefore, we see here an introduction into the facilitative role of tDCS experience enhancement that can potentially improve people’s skill level in order that the participant could reach the skill-challenge balance [133] that allows for a greater movement into flow states [140]. Other transcranial stimulation technologies may be worth considering for future research on flow that may also show a facilitative effect such as transcranial alternating current stimulation (tACS) which stimulate at a specific frequency and has shown to result in entrainment of neural networks to improve cognitive performance such as spatial reasoning [141] and working memory [142].

## 4. Conclusions

This collection of results from the literature begins to show what type of elements in a person’s professional environment need to be taken into consideration to help facilitate flow along with an understanding of the importance of supporting a transference of skills from explicit knowledge systems to implicit procedural systems. The literature appeared to highlight the role of the BG and its related components as a key research direction in which to further explore to enable a greater facilitation of skill automaticity that appears highly related to flow state.

Furthermore, there appears to be support in the literature for a particular neurocognitive activity pattern for flow induction in which expertise and flow studies appear to show a hemispheric shift away from the frontal left evidenced by a resulting reduction of left frontal activity and an increase in frontal alpha while facilitating a greater allocation of neuronal resources to the visual-spatial processes of the right brain, thus resulting in higher levels of performance. Recent interventions such as tDCS have been shown to have a positive effect on the facilitation of flow states that have followed this pattern and with more research may prove to be an effective intervention for real life applications as they are low cost, safe and non-invasive. Due to their simple application, it may be possible to conceive a work environment in which people are working at high levels of productivity with low levels of distractibility from low voltage of electricity.

## Figures and Tables

**Table 1:** Nine components associated with the flow state experience [9] (Csikszentmihalyi, 1990).

1. Clear goals (expectations and rules are discernible, and goals are attainable and align appropriately with one’s skill set and abilities).
2. High level of concentration, a high degree of concentration on a limited field of attention (a person engaged in the activity will have the opportunity to focus and to delve deeply into it).
3. A loss of the feeling of self-consciousness, the merging of action and awareness.
4. Distorted sense of time, one’s subjective experience of time is altered.
5. Clear and immediate feedback (successes and failures in the course of the activity are apparent, so that behavior can be adjusted as needed).
6. Balance between skill level and challenge (the activity is neither too easy nor too difficult).
7. A sense of personal control over the situation or activity.
8. The activity is intrinsically rewarding, so there is an effortlessness of action.
9. People become absorbed in their activity, and the focus of awareness is narrowed down.
